# Experimental neurotransplantation treatment for hereditary cerebellar ataxias

**DOI:** 10.1186/s40673-016-0045-3

**Published:** 2016-04-04

**Authors:** Jan Cendelin

**Affiliations:** Laboratory of Neurodegenerative Disorders, Biomedical Center, Faculty of Medicine in Pilsen, Charles University in Prague, Alej Svobody 76, 323 00 Pilsen, Czech Republic

**Keywords:** Cerebellum, Hereditary cerebellar degeneration, Neurotransplantation

## Abstract

Hereditary cerebellar degenerations are a heterogeneous group of diseases often having a detrimental impact on patients’ quality of life. Unfortunately, no sufficiently effective causal therapy is available for human patients at present. There are several therapies that have been shown to affect the pathogenetic process and thereby to delay the progress of the disease in mouse models of cerebellar ataxias. The second experimental therapeutic approach for hereditary cerebellar ataxias is neurotransplantation. Grafted cells might provide an effect via delivery of a scarce neurotransmitter, substitution of lost cells if functionally integrated and rescue or trophic support of degenerating cells. The results of cerebellar transplantation research over the past 30 years are reviewed here and potential benefits and limitations of neurotransplantation therapy are discussed.

## Background

Hereditary cerebellar ataxias are neurodegenerative diseases usually having a detrimental impact on patients’ quality of life or even leading to premature death. Patients suffer from a cerebellar syndrome, also combined in most of the hereditary ataxias with extracerebellar neurological symptoms, and in some cases other organ systems are also affected (for review see [1–5]).

Unfortunately, no sufficiently effective causal therapy is available for human patients at present (for review see [6, 7]). Therapy is mostly symptomatic. Common therapies which can help to delay complete disability in the patient are rehabilitation and physiotherapy [7]. Pharmacotherapy allows mitigation of some particular or associated symptoms [6, 7]. Nevertheless, these therapies do not influence progression of the degeneration. Only in some of the ataxias specific treatment is available. For instance, ataxia with vitamin E deficiency (AVED) responds to supplementation with vitamin E [8] and, analogously, autosomal recessive cerebellar ataxia type 2 (ARCA-2) might potentially be treated with coenzyme Q10 [9]. In general, there is no therapy which would be effective to completely stop or prevent progression of the degeneration or restore lost cerebellar function in hereditary cerebellar ataxias.

Hereditary ataxias are heterogeneous from the point of view of the genetics, pathogenesis and manifestations at both morphological and functional level. In many of them, the molecular mechanisms of pathogenesis are not known. Thus, development of causal therapy is difficult and there might not be any universal approach applicable for all of them. Several experimental studies in laboratory animals have already succeeded in targeting pathogenetic processes, and neurotransplantation research has also shown some promising results (for overview see Table 1). Nevertheless, these therapies are still under investigation and none of these approaches has yet become a routine method for human therapy. Such research, however, did not aim only to contribute to development of potential therapies but also to improve knowledge about the pathogenesis of hereditary ataxias. The results of cerebellar transplantation research over the past 30 years will be reviewed here.

**Table 1:** Experimental therapeutic approaches for cerebellar degenerations

Therapy	Subject	Effect	References
Inhibition of mutated gene expression	SCA3 mice	-clearance of nuclear accumulation of ataxin-3 -improved motor performance -alleviation of neuropathological changes	[14]
		-suppression of already manifested symptoms	[11]
	SCA7 mice	-reduction of ataxin-7 aggregation -improved motor performance -prevention of synaptic loss between climbing fibers and Purkinje cells	[13]
Enhancement of pathological protein elimination	SCA3 mice	-decrease of ataxin-3 accumulation -suppression of pontine neuronal death -improved motor performance	[15]
	SCA7 mice	-reduction of ataxin-7 intranuclear inclusions -improved motor performance	[12]
Stabilization of calcium signaling	SCA2, SCA3 mice	-alleviation of neuronal cell loss -improved motor performance	[17, 18]
Transcranial direct current stimulation	Human patients	-neuromodulation of the cerebellum	[19]
Embryonic (fetal) neural tissue transplantation	pcd mice	-colonization of the host molecular layer	[31–33, 35, 38]
		-improved motor performance	[40, 41]
	Lurcher mice	-organotypic organization of the graft, colonization of the host molecular layer	[54, 58, 59]
		-mild improvement of gait	[57]
	Weaver mice	-organotypic organization of the graft -colonization of the host cerebellum with granule-like cells	[64, 65]
	SCA1 mice	-improved motor performance	[80]
Neural precursor transplantation	Nervous mice	-support and rescue of host’s Purkinje cells -reduction of the excessive tissue plasminogen activator level -improved motor performance	[71, 72]
	Meander tail mice	-replenishment of deficient granule cell population	[76]
	SCA1 mice	-increased survival of Purkinje cells -improved motor performance	[81]
	SCA3 mice	-increased Purkinje cell survival -reduced granular layer atrophy -improved motor performance	[85]
	Niemann-Pick disease type C mice	-lengthened their life span without influencing the decline of motor performance	[92]
		-increased Purkinje cell survival -alleviation of neuropathological changes	[93]
	Niemann-Pick disease type A mice	-alleviation of neuropathological changes without influencing the decline of motor performance	[86]
Embryonic stem cell, embryonic stem cell-derived neural precursor transplantation	Weaver mice	-no adoption of region-specific cell identities	[67]
	Harlequin mice	-induction of endogenous neuronal precursor proliferation -delayed onset of the ataxia	[79]
Mesenchymal stem cell transplantation	Lurcher mice	-increased Purkinje cell survival -improved motor performance	[60]
	SCA1 mice	-mitigated cerebellar disorganization -improved motor performance	[82]
	SCA2 mice	-increased Purkinje cell survival -delayed disease onset -improved motor performance	[83]
	Niemann-Pick disease type C mice	-reduced astrocytic and microglial activation -increased Purkinje cell survival -improved motor performance	[87–90]

## Experimental therapeutic approaches targeting pathogenetic processes

For experimental investigation of potential therapies for hereditary degenerative cerebellar ataxias, as well as of their pathogenesis, cerebellar mutant mice have been widely used. There are numerous spontaneous mutants as well as transgenic mouse models of particular human diseases, both having some advantages and also limitations for their use and for translation of the findings into human medicine (for review see [10]).

Intervention into the pathogenic process seems to be an optimal strategy since it could theoretically prevent or delay development of degeneration and improve the functionality of remaining cells, if started in time. Several experimental studies targeting pathogenic processes have been done in transgenic mouse models carrying the human pathological allele causing cerebellar degeneration. These studies have shown not only a potential direction for the development of a specific therapeutic method but also confirmed the role of particular mechanisms in pathogenesis of the diseases [11–15].

In some hereditary cerebellar degenerations, a noxious effect of accumulated abnormal protein which is more resistant to proteolytic degradation is expected. Thus, silencing of the mutated gene’s expression might be helpful. In a mouse model of spinocerebellar ataxia type 3 (SCA3), halting the expression of ataxin-3 cleared nuclear accumulation of the abnormal protein in SCA3 mice [16]. Silencing of the expression of mutant ataxin-3 started after disease onset alleviated motor impairments as well as neuropathological changes [14]. Boy et al. [11] showed that turning off the expression of ataxin-3 at an early stage of the disease can even completely reverse already manifested symptoms in the mice. Similarly, in SCA7 mice, suppression of mutant allele expression by 50 %, started one month after the onset of ataxia, was effective at halting or even reversing motor symptoms, reducing mutant ataxin-7 aggregation in Purkinje cells and preventing synaptic loss between climbing fibers and Purkinje cells [13].

Another possibility is to enhance elimination of the pathological protein. Stimulation of proteasome activity by Rho-kinase, promoting mutant ataxin-3 degradation, alleviated the neurological phenotype in SCA3 mice [15]. Treatment with interferon beta reduced mutant ataxin-7 in intranuclear inclusions and led to improvement of balance and motor coordination in SCA7 mice [12].

Mutant ataxin-2 and ataxin-3 with expanded polyglutamine tracts have been shown to interact specifically with type 1 inositol 1,4,5-trisphosphate receptor (InsP3R1), an intracellular calcium (Ca^2+^) release channel, and sensitize it to activation [17, 18]. Treatment with dantrolene, a calcium ion stabilizer, alleviated motor deficits and neuronal cell loss in SCA2 and SCA3 transgenic mouse models [17, 18]. These findings suggest that disturbed calcium signaling may play a role in the pathogenesis of SCA2 and SCA3 [17, 18].

Recently, the possible therapeutic potential of non-invasive cerebellar stimulation with transcranial magnetic stimulation or transcranial direct current stimulation has been discussed [19, 20]. The stimulation is capable of influencing various cerebellar functions and thus it might become an applicable rehabilitation strategy; nevertheless, further studies are still necessary to validate this therapeutic approach [20].

Taken together, it has been shown in mouse models of hereditary cerebellar degeneration that it is possible to suppress the degenerative process and thereby prevent or delay irreversible neuropathological changes and to improve or stimulate residual cerebellar function. Some of these methods are based on genetic modifications and thus require a thorough assessment of potential risks prior to application in humans. Others are based just on drug administration and are therefore more easily acceptable to patients. The problem of these approaches, however, is that they cannot solve advanced cell loss and thus they would probably be ineffective in functional recovery when severe cell loss has already occurred. Neurotransplantation therapy, on the other hand, aims primarily to restore cerebellar function by substitution of cells that have been already lost rather than to prevent damage of the cerebellum. Nevertheless, it has subsequently been shown that it can take effect in various ways.

## Neurotransplantation in cerebellar mutant mice

Cerebellar transplantation has been investigated intensively for a long time using ataxic mice, both spontaneous mutants and transgenic models. Beneficial effects have been shown in some cases. Nevertheless, some findings are controversial and by now it is not clear whether neurotransplantation could become a routine therapy for cerebellar degeneration in humans, and there are still serious limitations. The problems of stem cell- and neurotransplantation-based therapy have already been reviewed by Rossi and Cattaneo in 2002 [21]. Most of the issues they pointed out, however, persist to this day.

For experimental neurotransplantation, various types of cell can be used: embryonic (fetal) neural tissue, embryonic (fetal) or adult neural stem cells, embryonic stem cells, adult stem cells, mesenchymal stem cells isolated from various tissues (e.g. bone marrow, adipose tissue), and carcinoma stem cells that are considered as dangerous for the risk of tumorigenesis. All these cells have some advantages and disadvantages dealing with biological as well as ethical aspects, which would become of much higher importance in humans. These various grafts have been transplanted into various cerebellar mutant mice and thus we have many but not all pieces of a large and diverse mosaic.

**Purkinje cell degeneration (pcd) mice** suffering from almost complete loss of Purkinje cells, slowly progressive granule cell degeneration, moderate reduction of cerebellar nuclei and some extracerebellar disorders [22–29] have been shown to be a good model for neurotransplantation research and they were also used for pioneering studies by the teams of Constatino Sotelo [30–36] and Lazaros C. Triarhou [37–41]. In these mice, solid embryonic cerebellar grafts formed a characteristic three-layered organization with the presence of parallel fibers which were, however, not oriented in a parallel fashion and were reduced in number compared with the normal cerebellar cortex [37]. Grafted Purkinje cells were able to migrate from the solid embryonic cerebellar graft, and cells from both solid and dissociated grafts colonized the host molecular layer, albeit being localized also in its superficial parts [31–33, 35, 38]. Sotelo and Alvarado-Mallart [33] reported that such neuronal invasion is a selective process since only Purkinje cells were able to penetrate into the molecular layer of the host cerebellum. They have also suggested that the deficient molecular layer of pcd mice exerts a selective neurotrophic effect on neurons of the missing category, and that the embryonic neurons are able to respond to these signals [32]. Carletti and Rossi [42] also found that the pcd cerebellum provides signals inducing selective mechanisms that favor the survival of donor Purkinje cells.

Grafted Purkinje cells synaptically integrated within the host cerebellar cortex, having synaptic contacts like normal Purkinje cells but lacking basket fiber-pinceau formations around the initial segment of the axon [30, 33, 35]. Intracellular recordings from grafted Purkinje cells located in the molecular layer revealed normal bioelectrical properties and demonstrated that grafted cells responded to stimulation mediated through both climbing and mossy fibers as well as to inhibitory signals [34]. On the other hand, Rosenfeld et al. [43] reported that grafted cells formed normal cerebellar cytoarchitecture only with other grafted cells and not with the cells of the cerebellum of host pcd mice. Furthermore, re-establishment of the corticonuclear projections in pcd mice is problematic [35]. The granule cell layer has been shown to prevent grafted cells from migrating and their axons from growing toward the deep cerebellar nuclei [44]. Thus, proximity of the cerebellar nuclei is necessary to establish projections of grafted Purkinje cells to the nuclei [31, 39, 45] since Purkinje cells localized in their normal position at the border of the granule and molecular layer have a limited possibility of contacting the cerebellar nuclei [44]. Triarhou et al. [39] reported that, if grafted onto the deep cerebellar nuclei, Purkinje cells are capable of forming axonal contacts in the cerebellar nuclei and then of moving to the cortical locality. In pcd mice, Sotelo et al. [36] showed that, for grafted Purkinje cell radial migration, the host’s Bergmann fibers were occupied and that grafted embryonic Purkinje cells can trigger molecular changes in the Bergmann fibers necessary for their migration.

Bilateral transplantation of a fetal cerebellar cell suspension into the deep cerebellar nuclei partially restored corticonuclear projections in the cerebellum and improved motor coordination and increased locomotor activity of pcd mice [40, 41].

Intravenous injection of bone marrow-derived cells at the age of 20 days led to reduction of the degenerative process in the olfactory bulb and improvement of olfaction in pcd mice, but did not change the degeneration of cerebellar Purkinje cells [46].

**Lurcher mutant mice** lose virtually all Purkinje cells and their granule cells, other cerebellar cortex interneurons and inferior olive neurons are substantially reduced as a secondary consequence, while cerebellar nuclei are less affected [47–51]. Primary degeneration of Purkinje cells is caused by the *Grid2*^*Lc*^ mutation in the δ2 glutamate receptor (GluRδ2) encoding gene [52]. GluRδ2 receptors are expressed at high levels in cerebellar Purkinje cell bodies and dendrites and are important for function of their synapses within the molecular layer [53]. The *Grid2*^*Lc*^ mutation is a gain of function mutation, which changes the receptor into a leaky membrane channel that chronically depolarizes the cells inducing their degeneration [52].

In these mice, embryonic cerebellar grafts have been also studied several times. Tomey and Heckroth [54], who grafted a cerebellar cell suspension, reported 50 % graft survival in both young and adult Lurcher mice. Later cell suspension and solid cerebellar graft survival over 60 % has been found two and six months after transplantation in Lurchers and the survival rates were comparable to those in wild type mice [55–57]. Aggregates of grafted cells with organotypic organization on the surface of the host cerebellum and invasion of grafted cells into the host’s molecular layer have been observed [54, 58, 59]. Nevertheless, migration of grafted cells or fiber sprouting to locations distal from the engrafting place, e.g. to the cerebellar nuclei, were rare in Lurchers [56, 57]. Timely transplantation of normal embryonic Purkinje cells can prevent secondary degeneration of cerebellar granule cells and inferior olive neurons, and this provides evidence for the secondary nature of degeneration in these cell categories and indicates at least some level of integration of the graft within the host cerebellar cortex [59]. Only a mild effect of embryonic cerebellar cell suspension on gait parameters, insufficient from a therapeutic point of view, has been observed in adult Lurcher mice [57].

A significant improvement in performance on motor tests was observed by Jones et al. [60] after transplantation of mesenchymal stem cells into the cerebellum of newborn Lurcher mice. Donor cells were located adjacent to the Purkinje cell layer and produced neurotrophic factors (BDNF, NT-3 and GDNF) that increased Purkinje cell survival [60]. The question, however, is whether the Lurcher Purkinje cells, still having their mutated δ2 receptor [52], are fully functional and whether their survival is the sole reason for the functional effect of the transplantation.

**Weaver mice** carry the missense *Grik*^*Wv*^ mutation of the gene encoding a G-protein coupled with inward rectifying potassium channel [61]. They suffer from disorganization of granule layer development and degeneration of cerebellar granule cells [62, 63]. In Weaver mice, dissociated embryonic cerebellar cells grafted onto the cerebellum developed into a large mass of tissue with a trilaminar organization [64–66]. Grafted granule-like cells proliferated, migrated from the place of transplantation and were able to develop synaptic contacts in the Weaver mutant cerebellum [64–66].

In Weaver mutant mice, grafted cerebellar-derived multipotent astrocytic stem cells and embryonic stem cell-derived neural precursors were not capable of adopting region-specific cell identities, but the milieu of the granuloprival host cerebellum provided signals slightly supporting neuronal differentiation of donor cells [67].

**Nervous mutant mice** are characterized by mitochondrial abnormalities and degeneration of Purkinje cells [68]. The degeneration is mediated by tissue plasminogen activator, levels of which are elevated in the cerebellum of homozygous Nervous mice [69, 70]. Li et al. [71] have shown that undifferentiated neural stem cells grafted into the cerebellar cortex of newborn Nervous mice contacted the host’s Purkinje cells and supported their mitochondrial function, dendritic growth and synaptogenesis, rescued the cells from degeneration and improved motor coordination. Also, in organotypic slices of Nervous cerebellum, more Purkinje cells survived when cocultured with neural stem cells in vitro [71]. The rescue of Purkinje cells was related to reduction of the excessive tissue plasminogen activator level mediated by the neural stem cell-induced rectification of tissue plasminogen activator gene expression [71]. Thus, in this case, grafted cells modulated the pathogenetic pathway of Purkinje cell degeneration. Rescue of Purkinje cells and motor performance by grafted neural stem cells that did not fully differentiate into neurons but formed gap junctions with the host’s cells have also been reported in Nervous mice by Jaderstad et al. [72].

**Meander tail mice** are recessive mutants showing ankylosis or fusion of tail vertebrae [73] and defective development of the cerebellum resulting in abnormal arrangement of cerebellar lobes, reduced number of granule cells, random positioning of Purkinje cells and disorganized spinocerebellar projection, most evident in the anterior lobes [74, 75]. Rosario et al. [76] grafted multipotent neural progenitor cells isolated from the external granular layer of the cerebellum of newborn wild type mice into the granuloprival area of the cerebellum of newborn Meander tail mice. Most of the grafted cells differentiated into granule cells and thus partially replenished the deficient neuronal population [76]. In this case, donor neural stem cells were derived from a structure which is the natural source of the cell type that had to be substituted and the donor was of the same ontogenetic period as the host. Nevertheless, some factors that theoretically force the progenitors to have a potential for multiple fates to differentiate toward the repletion of deficient cell types might be supposed [76].

**Harlequin mutant mice** are a model of oxidative stress-mediated cerebellar degeneration and ataxia [77, 78]. Human embryonic stem cell-derived progenitors of cerebellar granule cells grafted into the cerebellum of Harlequin mice survived only for several weeks, but induced proliferation of endogenous neuronal precursors in the leptomeninges that crossed the molecular layer and differentiated into mature neurons [79]. Furthermore, the treatment preserved granular and Purkinje cell layers and delayed onset of the ataxia as measured with the rotarod test [79].

Several strains of transgenic mice serve as models of particular human autosomal dominant **spinocerebellar ataxias (SCAs)**. In **SCA1 mice**, a transgenic mouse model of human spinocerebellar ataxia type 1, transplantation of an embryonic cerebellar cell suspension at the time of ataxia onset led to improvement in motor function as compared with sham-operated controls [80]. The grafts were found in various locations, some intraparenchymally while others formed extraparenchymal structures [80]. Nevertheless, most of the grafts survived for up to 20 weeks after engraftment and contained donor-derived Purkinje cells [80].

Chintawar et al. [81] grafted neural precursor cells into the cerebellar white matter of SCA1 mice at the age of absent, initial and significant Purkinje cell loss. Only in mice with significant degeneration did grafted cells migrate into the cerebellar cortex and the treatment lead to improvement of motor skills [81]. Despite grafted cells not adopting the Purkinje cell phenotype, the cerebellar cortex of treated mice contained more surviving Purkinje cells which, contrary to those in sham-operated SCA1 mice, showed normalization of membrane potential [81]. Since no marked increase in levels of neurotrophic factors had been detected, Chintawar et al. [81] suggested that the protective effect of grafted neuroprogenitor cells on host Purkinje neurons was mediated rather by direct contact between grafted and host cells. Similar findings were reported by Jaderstad et al. [72].

Intrathecal injection of mesenchymal stem cells mitigated cerebellar disorganization, suppressed atrophy of Purkinje cell dendrites and normalized motor coordination deficits in SCA1 mice [82].

Chang et al. [83] investigated the effect of intravenous (via the tail vein) or intracranial (via the occipital foramen magnum) injection of human mesenchymal stem cells in **SCA2 transgenic mice**. After intravenous injection, grafted cells were detected in the cerebellar white matter, molecular layer, in the lumen of blood vessels in the white matter and in the cerebral cortex [83]. In mice treated this way, increased survival of host Purkinje cells, delayed onset of disease and improved motor function has been observed [83]. In the case of intracranial injection, grafted cells were seen only in a few lumens of blood vessels, but not inside the cerebellar tissue, and this approach failed to achieve the neuroprotective effect observed after intravenous administration [83].

**SCA3 (Machado-Joseph disease)** is a polyglutamine disorder in pathogenesis of which inflammatory processes play a role [84]. In a mouse model of SCA3, cerebellar neural stem cells isolated from neonatal wild type mice and cultured and differentiated in vitro were transplanted into the cerebellum [85]. Grafted neural stem cells differentiated into astrocytes and oligodendrocytes as well as into functional neurons [85]. The treatment alleviated motor impairment, reduced Purkinje cell loss, granular layer atrophy and mutant ataxin-3 aggregates [85]. The transplantation has been also suggested to suppress neuroinflammation since it reduced the levels of inflammatory modulators interleukin-1b, interleukin-6 and tumor necrosis factor α in SCA3 mice [85]. Furthermore, the transplantation induced elevation of BDNF level in these mice [85].

**Niemann-Pick disease** is an autosomal recessive lysosomal storage disease with accumulation of sphingomyelin in many tissues. Niemann-Pick disease type C is a defect of sphingomyelin transport and typically affects the cerebellum. Niemann-Pick disease types A and B are caused by defects of sphingomyelinase enzyme. Unlike human patients, Niemann-Pick type A mice suffer from progressive degeneration of Purkinje cells in the cerebellum and develop ataxia [86].

In a mouse model of Niemann-Pick disease type C, therapy with mesenchymal stem cells has been tested with promising results [87–89]. Bone marrow-derived mesenchymal stem cell transplantation reduced astrocytic and microglial activation in the cerebellum of these mice [87], led to an increase in Purkinje cell numbers and improved motor skills [88]. It has been shown that electrically active Purkinje neurons originated from existing Purkinje cells through fusion-like events with grafted mesenchymal stem cells [88]. Lee et al. [90] showed that bone marrow-derived mesenchymal stem cells promote the survival of Purkinje cells in Niemann-Pick disease type C mice by correction of the altered calcium homeostasis, modulation of sphingolipid metabolism and by inhibition of apoptotic pathways. Also, transplantation of adipose tissue-derived stem cells rescued Purkinje neurons, alleviated inflammatory responses and restored motor coordination in Niemann-Pick disease type C mice [89].

In a mouse model of Niemann-Pick disease, bone marrow-derived cells were also used for ex vivo gene therapy [91]. Normal murine bone marrow cells were genetically modified to overproduce human acid sphingomyelinase, the enzyme which is defective in Niemann-Pick disease types A and B, and were injected intravenously at the age of 3 days and intracerebrally at 4 weeks to sphingomyelinase-deficient mice [91]. The treatment led to transient normalization of enzyme activity and reduction of sphingomyelin in the tissues, suppression of Purkinje cell degeneration and normalization of cerebellar function [91].

Injection of neural stem cells into the cerebellum of Niemann-Pick disease type C1 mice lengthened their life span, but did not influence the decline of motor performance as measured by the rotarod test [92]. Similarly, transplantation of human neural stem cells into neonatal mice with targeted deletion of the sphingomyelinase gene, a model of Niemann-Pick type A, did not prevent Purkinje cell loss or decline in rotarod performance, despite reduction of histopathological changes that were even more widespread than the distribution of grafted neural stem cells [86]. On the other hand, Lee et al. [93] reported alleviation of neuropathological features, an increase of surviving Purkinje cells and enhancement of neuronal networks with functional synaptic transmission in Niemann-Pick disease type C mice treated with intracerebellar injection of conditionally immortalized neural stem cells of hippocampal origin.

## Mechanisms of the effect of the grafts

Experiments in models of neurological diseases including cerebellar degeneration have shown that grafts can employ their effects via various mechanisms. One relatively simple way is **delivery of a scarce neurotransmitter**. The most successful neurotransplantation therapy used in human patients is that for Parkinson’s disease. Rossi and Cattaneo [21] pointed out that in this case dopaminergic neurons of the *substantia nigra* are grafted directly onto the striatum which is the target structure for the dopaminergic projection, and that paracrine release of dopamine is sufficient to restore function without the need to reestablish specific cell-to-cell projections. Furthermore, fetal *substantia nigra* directly provides the desired cell phenotype. Thus the nature of Parkinson’s disease represents an optimal situation to be solved by substitution of lost neurons since the task of the graft is just to deliver an adequate amount of a neurotransmitter, in this case dopamine.

Theoretically, **complete substitution of lost cells** and precise reconnection of neural circuitry by grafted cells would be optimal, since it would completely restore normal structure and function. However, this is probably the most difficult endpoint to achieve since it requires sufficient numbers of surviving grafted cells, migration into appropriate positions, differentiation into appropriate cell types, fiber sprouting and establishment of precise cell-to-cell connections (for review see [21]). The cerebellum represents a structure for which specific topographically arranged projections with other structures, but also connections within the cerebellar cortex as well as corticonuclear projections, are essential for its function [21]. A good source of cerebellar cell phenotypes to substitute lost cells is embryonic (fetal) cerebellar tissue [31–33, 35, 38, 56, 58, 65, 80, 94]. Nevertheless, functional integration of this kind of graft is not easy (see above). Neural precursors, on the other hand, have mostly been shown to fail in adopting the specific cerebellar phenotypes to be substituted or in completing neuronal differentiation at all [71, 72, 81, 86] despite the fact that they are able to migrate into the cerebellum even after injection into the lateral ventricle [95]. The differentiation of these cells might depend on both the local niche and their origin. For instance, Klein et al. [96] showed that neural stem cells from the forebrain and the cerebellum fail to generate neurons when grafted ectopically, but neural stem cells of cerebellar origin acquired features of cerebellar granule cells when grafted onto the perinatal cerebellum. Also, adult highly purified hematopoietic cells have been shown to fail to transdifferentiate into neurons when injected into the normal mouse cerebellum [97]. On the other hand, small number of donor derived Purkinje cells has been found in the cerebellum of mice 15 months after intravenous injection of bone marrow-derived cells [98] (see also below).

Many studies have shown that grafts can provide a **neuroprotective effect, trophic or metabolic support** for degenerating cells, or modulate the inflammatory response participating in cerebellar damage. In this way the graft may rescue the cells or delay degeneration if applied before the changes become irreversible. These effects have been suggested mainly in mesenchymal stem cells that have been shown to produce neurotrophic factors rescuing degenerating Purkinje cells [60] and to modulate glial activation, inflammation and apoptosis activation [87, 89, 90]. Neural precursors have been suggested to prevent degeneration of the host’s Purkinje cells by making direct contact with them, mediating metabolic or trophic support [71, 81]. Jaderstad et al. [72] demonstrated the importance of gap junctions for interactions between grafted neural stem cells and host Purkinje cells. They found that functional gap junctions were formed between grafted neural stem cells and host cells at risk in Nervous and SCA1 mice [72]. Gap junction formation was associated with rescue of Purkinje cells from degeneration and with improvement of motor performance, and these beneficial effects of neural stem cells were abrogated when formation or function of the gap junctions was inhibited [72].

Grafted bone marrow-derived cells have been shown to give origin to Purkinje cells [98, 99] which in some cases have arisen from **cell fusion** between the grafted cells and host Purkinje cells [100, 101]. Fusion-like processes may rescue Purkinje cells from degeneration [88]. On the other hand, Nern et al. [102] suggested that fusion of hematopoietic cells with Purkinje cells is only a transient phenomenon and does not establish stable heterocaryons under physiological conditions. Nevertheless, the frequency of fusion between bone marrow-derived cells and Purkinje neurons can be increased by pathological processes, e.g. inflammation or mild degeneration [101–103].

Mesenchymal stem cells have also been used as a tool for **ex vivo gene therapy** in a mouse model of Niemann-Pick disease [91, 104]. Normal, genetically unmodified neural stem cells even rectified abnormal gene expression thereby disrupting the pathogenetic process [71].

The cerebellum can be also affected by demyelinating processes. In this case, transplantation of cells providing **remyelination** might be helpful. In a rat model of radiation-induced demyelination causing cognitive and motor coordination impairments, injection of human embryonic stem cell-derived oligodendrocyte progenitors into the central cerebellar white matter led to remyelination and improved motor coordination measured using the rotarod test [105].

## Conclusion

Several clinical studies have reported successful use of cerebellar transplantation or systemic administration of stem cells in degenerative diseases that affect the cerebellum in humans [106–108]. Donor-derived astrocytes as well as neurons including Purkinje cells have been found in the cerebella of patients after bone marrow transplantation [99, 109]. Nevertheless, this approach is still rather in the stage of experiments in laboratory animals, and reliable evidence for long-persisting efficiency and safety of neurotransplantation therapy for hereditary ataxias is still missing. A donor-derived brain tumor that developed from grafted neural stem cells has been reported in a patient with ataxia telangiectasia [110]. Prior to routine use of cerebellar transplantation in human patients, if it will ever be possible, it would be necessary to answer many questions regarding the long-term efficiency of the therapy and also its safety.

Proliferation, migration, differentiation and fiber sprouting of grafted cells that are necessary for cell replacement and full functional integration strongly depend not only on the properties of the cells but also on the host tissue niche. Neurogenic features of the host tissue are determined by normal features of the tissue, in particular brain structure, and by pathological changes of the diseased tissue. Some factors, like lack of a certain cell type, might promote appropriate development of the graft, while others could have a negative effect, limiting neuronal differentiation and integration of grafted cells (for review see [21]). The task for future research is elucidating all important factors which play a role in determination of the graft’s fate in particular diseases. Knowing these factors can help to predict the particular diseases in which neurotransplantation could be effective or to develop therapeutic interventions to make the host tissue niche more favorable for the development and functional integration of the graft.

The most effective source of cells having determined specific cerebellar phenotypes is embryonic cerebellar tissue. These cells have to be harvested from embryos and they cannot be expanded in vitro to sufficient amounts. Thus, obtaining grafts for clinical use would be problematic from an ethical point of view as well as that of quality and quantity. Nevertheless, it has been shown that induction of differentiation of embryonic stem cells into cerebellar Purkinje cells and granule cell precursors is also possible [111]. Since long-term and intensive proliferation of embryonic stem cells in vitro is easy, they could be a more acceptable type of graft if their efficient cerebellar differentiation in vivo could be induced. Adult stem cells obtained from various tissues would be an ethically acceptable material for transplantation therapy and, again, the technique of massive differentiation into cerebellar cell phenotypes has to be developed. Neuronal differentiation might be easier in neural stem cells. Neural stem cells isolated from the postnatal cerebellum and expanded in vitro as neurospheres have been shown to differentiate into astrocytes, oligodendrocytes as well as neurons after transplantation into the cerebellum in the form of single neurosphere [112]. When cultured as neurospheres they enable engraftment in the form of single cell suspension or solid neurospheres containing more cells and being analogy of solid embryonic grafts that might be more resistant to detrimental effects of non-neurogenic niche in the pathologically changed cerebellar tissue. Unlike many adult stem cells neural stem cells cannot be obtained without ethical problems. Another research task is to find optimal methods of nerve fiber guidance that would promote reconstruction of neural circuitry. Up to now, mesenchymal stem cell transplantation seems to be quite effective in rescuing degenerating neurons in cerebellar mutant mice. Nevertheless, this kind of therapy has to be applied in time. Early diagnosis based on genetic testing of family members helps in this regard.

Beyond the investigation of therapeutic approaches for neurological diseases, neurotransplantation also remains a tool for studying the mechanisms and factors playing a role in brain development, cell differentiation and migration, and neural plasticity and regeneration [96, 113].

## Abbreviations

ARCA-2: autosomal recessive cerebellar ataxia type 2
AVED: ataxia with vitamin E deficiency
BDNF: brain-derived neurotrophic factor
GDNF: glial-cell line derived neurotrophic factor
InsP3R1: inositol 1,4,5- trisphosphate receptor
NT-3: neurotrophin 3
SCA: spinocerebellar ataxia
